# Development of ultrahigh‐performance liquid chromatography/mass spectrometry and ultrahigh‐performance supercritical fluid chromatography/mass spectrometry assays to determine the concentration of Bitrex™ and sodium saccharin in homemade facemask fit testing solutions

**DOI:** 10.1002/rcm.8848

**Published:** 2020-07-16

**Authors:** Julie M. Herniman, G. John Langley

**Affiliations:** ^1^ School of Chemistry, Faculty of Engineering and Physical Sciences University of Southampton, Highfield Southampton SO17 1BJ UK

## Abstract

Fast and easily transferable chromatography/mass spectrometry assays were required to detect and quantify the amount of Bitrex™ and sodium saccharin in homemade facemask fit testing solutions.

**Methods:**

Bitrex™ solutions were analysed using reversed‐phase ultrahigh‐performance liquid chromatography coupled with positive ion electrospray ionisation mass spectrometry (UHPLC/ESI‐MS). Separation was achieved using a mobile phase gradient with an Acquity BEH C18‐packed column. Sodium saccharin solutions were analysed using ultrahigh‐performance supercritical fluid chromatography coupled with negative ion electrospray ionisation (UHPSFC/ESI‐MS). Separation was achieved using isocratic elution with an Acquity UPC^2^ Torus Diol packed column and a methanol (25 mM ammonium acetate) co‐solvent.

**Results:**

The calibration curves obtained using the ratio of the active compound to an internal standard generated linear regression values (R^2^) >0.99. Samples analysed prior to and after an autoclave sterilisation process and bottling gave repeatable measurements within 10% of the expected concentration.

**Conclusions:**

The two assays afford a fast robust and quantitative analytical method for the detection of the active components used to test the efficacy of the homemade facemask testing solutions.

## 1 INTRODUCTION

**FIGURE 1:**
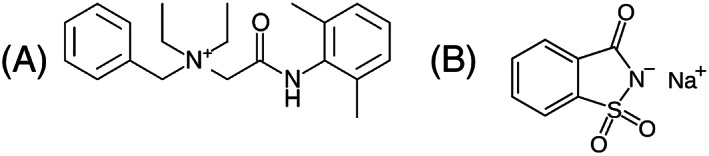
Chemical structures of A, Bitrex™ (denatonium benzoate) and B, sodium saccharin

In extreme environments where airborne pollutants may be present, e.g. toxic gases, fumes, vapours, and other harmful pollutants, it is essential that personal protective equipment is worn and fitted correctly. In hospitals and care homes the use of respirators and face protection ensures that contaminants do not cause potential harm to the wearer. Filtering facepiece (FFP)1 masks work to protect individuals from spreading their own saliva and bodily fluids into the wider environment, while FFP2 masks are respirator face masks that filter out noxious and toxic chemicals/odours, vapours, and other harmful pollutants from the air.^1^ All protective equipment must be fitted correctly and adjusted individually to the wearer's face. A Face Fit Test is normally undertaken prior to the respirator or facemask being worn and the fit needs to be checked regularly. There are two tests as defined by the Occupation Health and Standard Association (OSHA) in 29 CFR 1910.134.^2^ The Qualitative Fit Test (QLFT) is a simple pass/fail test that measures the user's response to a test solution. The Quantitative Fit Test (QNFT) is an assessment of the adequacy of fit of the respirator by measuring the amount of leakage into the respirator.^3^ Two different solutions are commonly used to assess disposable and reusable half‐masks: denatonium benzoate, otherwise known by the brand name Bitrex™,^4^ and sodium saccharin^5^ (for structures, see Figure 1). The recent outbreak of COVID‐19 in the UK has led to shortages of these commercial spray solutions, as demand has significantly increased in line with increased usage of masks and respirators. Chemists at the University of Southampton have produced homemade solutions^6^ following a method adapted from Fakherpour et al^7^ and made to the British Standard BS ISO 16975‐3:2017^8^ and the US standard set out by the OHSA in 29 CFR 1910.134.^2^

An analytical quality control (QC) method was required to speedily assess the quantity of taste test compound in each prepared batch solution. This was required immediately after batch preparation and following an autoclave sterilisation (>120°C) and subsequent bottling process. A minimum of 10% of the bottles were tested prior to distribution.

The initial aim of the analytical QC methodology was to develop fast, easily transferable, fit‐for‐purpose tests that could be undertaken across many analytical laboratories. A reversed‐phase (RP) high‐ or ultrahigh‐performance liquid chromatograph is one of the most common instruments available to a QC laboratory; hence, this separation technique coupled to mass spectrometry was initially attempted for the analysis of both solutions. The methods used were adapted from several previously published articles.^9^, ^10^, ^11^, ^12^, ^13^, ^14^

As Bitrex™ is a quaternary ammonium compound positive ion electrospray ionisation (ESI) is required for its analysis, while negative ion ESI is required for the analysis of the sodium saccharin salt. Although RP‐UHPLC/MS methods were successfully developed for both Bitrex™ and sodium saccharin, an alternative chromatographic technique, ultrahigh‐performance supercritical fluid chromatography/mass spectrometry (UHPSFC/MS), was also investigated for both assays. This would maximise usage of available instrumentation at the University of Southampton, Chemistry Mass Spectrometry Laboratory. The two QC assays could then be run in parallel and both assays could also be undertaken on either instrument. Here the optimum chromatography and mass spectrometry methods are reported for each assay (RP‐UHPLC/MS for Bitrex™ and UHPSFC/MS for sodium saccharin). In its simplest form UHPSFC can be considered as a surrogate normal‐phase chromatography technique. Supercritical carbon dioxide (scCO_2_) is the primary mobile phase used and is a supercritical fluid above its critical point of 31.1°C and 73.8 bar, where it has properties intermediate between those of a gas and a liquid.^15^, ^16^, ^17^, ^18^ For many reasons scCO_2_ is the most commonly used mobile phase; its critical point is easily obtainable, and it is readily available, inexpensive, considered green, and relatively safe to use.^19^

UHPSFC can be easily coupled to a mass spectrometer using an atmospheric pressure ionisation source with a flow splitter. A make‐up solvent, such as methanol with formic acid, is delivered to the mass spectrometer *via* a splitter configuration to promote ionisation and ensure a stable spray.

## 2 EXPERIMENTAL

### 2.1 Chemicals

Acetonitrile, methanol, water (LC/MS grade) and formic acid (FA) were purchased from Thermo Fisher Scientific (Loughborough, UK). Denatonium benzoate, oxybutynin chloride, vanillic acid and ammonium acetate were purchased from Sigma‐Aldrich (Gillingham, UK). Sodium saccharin was obtained from Vimto Soft Drinks‐Nichols plc (Newton‐Le‐Willows, UK) and Nutraceuticals Group Europe (Redhill, UK) and used without further purification. Food grade carbon dioxide was purchased from BOC Special Gases (Manchester, UK).

### 2.2 Stock solutions

The standard compounds (Bitrex™ and sodium saccharin) were prepared volumetrically at a concentration of 10 mg made up to 10 mL (methanol) to give stock solutions of 1 mg/mL.

### 2.3 Internal standard preparation

**FIGURE 2:**
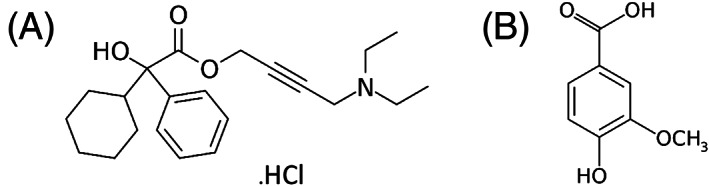
Chemical structures of internal standards: A, oxybutynin chloride and B, vanillic acid

The internal standard compounds (oxybutynin chloride and vanillic acid) were initially prepared at a concentration of 1 mg/mL in methanol and then diluted using volumetric dilution to the appropriate concentration (for structures, see Figure 2).

### 2.4 Calibration preparation

Standard calibration solutions were prepared volumetrically for Bitrex™ containing nominally 20, 50, 100, 150 and 200 ng/mL with oxybutynin chloride as the internal standard at 100 ng/L. Standard calibration curves were prepared for sodium saccharin containing nominally 5, 10, 15, 20, 32.5 and 50 μg/mL with vanillic acid as the internal standard at 10 μg/mL. The concentration of the standards was selected to ensure that the calibration curves correspond to the linear ionisation response region of each instrument.

### 2.5 Sample preparation

Each batch of Bitrex™ and sodium saccharin was prepared at two concentrations, one named as SENSE (to test the response of an individual) and one named as TEST (to test the facemask once it has been fitted to the individual). A volume of 1 mL of each batch solution was removed from the bulk and prepared for QC analysis. The Bitrex™ SENSE solution was prepared at a concentration of 135 mg/mL and this was serially diluted using methanol containing the oxybutynin chloride internal standard. The first step was a 1:1 dilution using the internal standard at 200 ng/mL followed by dilutions using the internal standard at 100 ng/mL to a concentration of 67.5 ng/mL. (×2000 dilution). The TEST solution concentration was 1.69 g/L and this solution was serially diluted using methanol containing the same internal standard to a concentration of 84.7 ng/mL (×20,000 dilution). The saccharin SENSE solution was prepared at a concentration of 8.3 mg/mL and was serially diluted using methanol containing the vanillic acid internal standard. The first step was a 1:1 dilution using the internal standard at 20 μg/mL followed by dilutions using the internal standard at 10 μg/mL to a concentration of 10.4 μg/mL (×800 dilution). The TEST solution concentration was 536 mg/mL and this solution was serially diluted using methanol containing the same internal standard to a concentration of 26.8 μg/mL (×20,000 dilution).

### 2.6 Chromatography

Separations were performed for the Bitrex™ samples using an Acquity ultrahigh‐performance liquid chromatograph with a BEH C18 column (1.7 μm particle size, 2 × 50 mm) (both from Waters, Wilmslow, UK). The column was held at 50°C in a column oven and 2.0 μL of each sample was injected. Solvent A, water + 0.2% FA, and solvent B, acetonitrile + 0.2% FA, were used for separation at a flow rate of 0.6 mL/min. A gradient elution was performed using the method reported in Table 1 and a 1‐min isocratic pre‐run was used for column equilibration.

**TABLE 1:** UHPLC gradient for the analysis of Bitrex™

Time (min)	Solvent A (%)	Solvent B (%)
0.00	80	20
1.30	0	100
1.35	0	100
1.50	80	20

Separations were performed for the sodium saccharin samples using an Acquity ultrahigh‐performance convergence chromatograph (UPC^2^, Waters) with a Torus Diol packed column (1.7 μm particle size, 3 × 100 mm; Waters). The column was held at 40°C in a column oven and 2.0 μL of each sample was injected. scCO_2_ with methanol (25 mM ammonium acetate) co‐solvent was used for separation at a flow rate of 1.5 mL/min. The scCO_2_ back pressure of the system was set to 150 bar. An isocratic elution was performed using co‐solvent B at 40% for 1.5 min.

### 2.7 Mass spectrometry

Positive ion ESI mass spectra were recorded using a triple quadrupole mass spectrometer (Xevo TQD) and an ESCi multi‐mode ionisation source (both from Waters) with the following conditions: capillary voltage 2.5 kV; cone voltage 20 V; extractor voltage 3.0 V; source temperature 150°C; desolvation temperature 600°C; desolvation gas flow rate 600 L/h (nitrogen). Acquisition and data processing were achieved using MassLynx™ version 4.1 and TargetLynx (Waters). Selected ion monitoring (SIM) was used for quantitation in preference to multiple reaction monitoring (MRM) so that the methods could easily be transferable to other instruments and laboratories. SIM was used to detect the molecular cation [M]^+^ for Bitrex™, nominal *m/z* 325, and the [M + H]^+^ ion for the internal standard oxybutynin chloride, nominal *m/z* 358, with a dwell time of 0.088 s.

Negative ion ESI mass spectra were recorded using a single quadrupole mass spectrometer (SQD 2) and an ESCi multi‐mode ionisation source (both from Waters) with the following conditions: capillary voltage 2.5 kV; cone voltage 20 V; extractor voltage 3.0 V; source temperature 150°C; desolvation temperature 500°C; desolvation gas flow rate 650 L/h (nitrogen). Acquisition and data processing were achieved using MassLynx™ version 4.1 and TargetLynx. An isocratic solvent manager (ISM) (Waters) was used to introduce the make‐up solvent, methanol (50 μM ammonium acetate), at a flow rate of 0.45 mL/min.

SIM was used to detect the deprotonated molecules [M − H]^−^ for saccharin, nominal *m/z* 182, and the internal standard vanillic acid, nominal *m/z* 167, with a dwell time of 0.163 s.

## 3 RESULTS AND DISCUSSION

### 3.1 Method development

**FIGURE 3:**
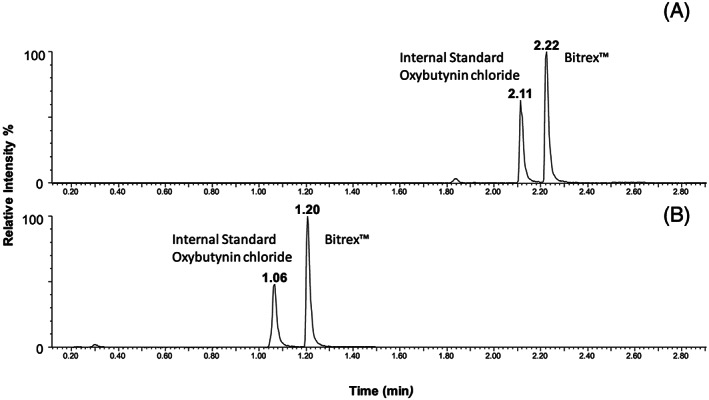
Reversed‐phase UHPLC/MS SIM chromatograms for oxybutynin chloride and Bitrex™ using A, 5‐min gradient and B, 1.5‐min gradient

**FIGURE 4:**
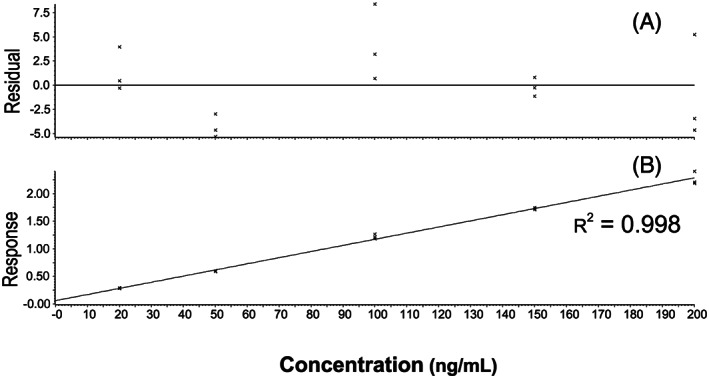
Calibration measurements for Bitrex™ using oxybutynin chloride as the internal standard: A, residuals and B, calibration curve produced using TargetLynx

**FIGURE 5:**
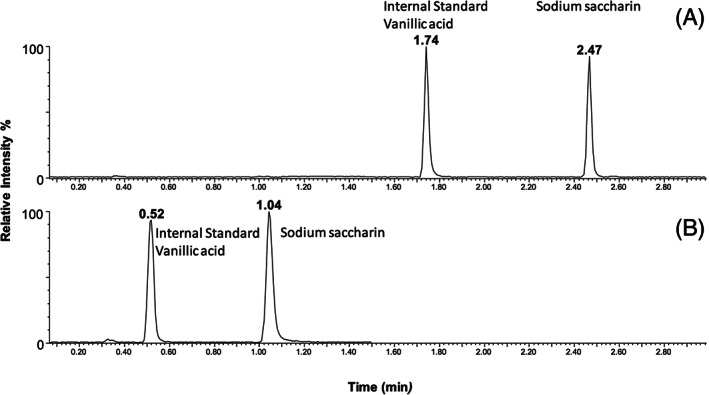
UHPSFC/MS SIM chromatograms for vanillic acid and sodium saccharin using A, 3‐min gradient and B, isocratic elution

In the first instance, commonly available RP‐UHPLC/MS assays requiring minimal modification and customisation were used to facilitate the QC analysis for both compounds. Generic gradients were used, starting at 5% solvent B increasing to 100% solvent B over 5 min with a standard BEH C18 column. The first task was to identify a suitable internal standard for each assay. Oxybutynin chloride was selected for the Bitrex™ assay since this was readily available in the laboratory and is routinely used as a component of the system suitability test used for RP‐UHPLC/MS (for structure, see Figure 2A). It has a similar retention time to Bitrex™ and its positive ion electrospray ionisation efficiency closely matched that of Bitrex™. To accelerate the analysis, the generic gradient was optimised to 20% solvent B increasing to 100% solvent B in 1.5 min with a 1‐min isocratic pre‐run (Figure 3). The linear response for Bitrex™ using this assay was defined using solutions of standard Bitrex™ ranging from 1 ng/mL to 1000 ng/mL. SIM for the molecule was used to improve peak profile and ensure that the methods could be transferred to other analysers. Once the linear response region had been defined, calibration solutions were prepared (20–200 ng/mL) containing the internal standard at a concentration within the middle of the curve (100 ng/mL). A five‐point calibration curve was constructed automatically with TargetLynx using the ratio of Bitrex™ to the internal standard, and gave R^2^ values of >0.99 (Figure 4).The same assay was attempted for the analysis of sodium saccharin using negative ion ESI; however, the peak shape for sodium saccharin was initially poor. This was somewhat improved by changing to water as the internal standard diluent to give a fit‐for‐purpose assay. The saccharin analysis was developed using an in‐house UHPSFC/MS assay, 10% co‐solvent (methanol 25 mM ammonium acetate) increasing to 40% co‐solvent over 5 min using a Torus Diol column. Vanillic acid was selected as the internal standard for this assay since this was readily available in the laboratory (for structure, see Figure 2B). Its negative ion ESI efficiency closely matched that of sodium saccharin and it gave a similar good chromatographic peak shape. To accelerate the analysis, the assay was optimised using an isocratic 40% co‐solvent method for 1.5 min (Figure 5), hence removing the need for a pre‐run column equilibration method. The linear response for sodium saccharin using this assay was defined using solutions of standard sodium saccharin ranging from 1 μg/mL to 1,000 μg/mL using SIM for the deprotonated molecule, to improve the peak profile. Once the linear response region had been defined, calibration solutions were prepared (5–50 μg/mL) containing the internal standard at a concentration in the middle of the curve (20 μg/mL). A five‐point calibration curve was constructed automatically with TargetLynx using the ratio of sodium saccharin to the internal standard, and gave R^2^ values of >0.99.

### 3.2 Application of the methodology

Following preparation of the bulk solutions, 1 mL of solution was removed for QC analysis. A calibration curve was created with the standards over the linear response range of the instrument prior to analysis and this must give a R^2^ value >0.99 before proceeding. If the R^2^ value is <0.99, creation of the calibration curve must be repeated and it should be adjusted if necessary. Each solution was diluted with the pre‐prepared internal standard solution in methanol to the appropriate concentration (Bitrex™, 67.5 and 84.75 ng/mL SENSE and TEST, respectively; sodium saccharin, 10.4 and 26.8 μg/mL SENSE and TEST, respectively). These pre‐autoclave solutions were then measured in triplicate ensuring that a solvent blank was analysed between each sample to monitor and prevent carry‐over. The data were interpolated using the calibration curve with TargetLynx. If the required concentration was calculated to be 10–12% of the required concentration, the batch solution could proceed to the bottling and autoclave stage. Following the autoclave stage, a minimum of 10% of bottles were randomly selected and 1 mL of solution was removed for QC analysis. These solutions were diluted to the appropriate concentration using the internal standard solution in methanol. These samples were analysed in triplicate ensuring a solvent blank between each analysis to monitor and prevent carry‐over. The data were interpolated using the calibration curve with TargetLynx. If the required concentration was calculated to be within 10% of the required concentration, the bottled solutions were released for distribution. Tables 2 and 3 show typical data from one batch of each solution.

**TABLE 2:** Quality control measurements and calculations for one batch of Bitrex™ TEST and SENSE (5 L)

Bitrex™ batch	Bulk prepared conc. (g/L)	Diluted conc. (ng/mL)	Pre‐autoclave measured (ng/mL)	Pre‐autoclave calculated (g/L)	Post‐autoclave measured (ng/mL)	Post‐autoclave calculated (g/L)
TEST	1.69	84.7	75.5 ± 0.4	1.51	75.7 ± 5.5	1.51
SENSE	0.135	67.5	67.2 ± 0.9	0.134	62.9 ± 4.2	0.126

**TABLE 3:** QC measurements and calculations for one batch of sodium saccharin TEST and SENSE (5 L)

Sodium saccharin batch	Bulk prepared conc. (g/L)	Diluted conc. (μg/mL)	Pre‐autoclave measured (μg/mL)	Pre‐autoclave calculated (g/L)	Post‐autoclave measured (μg/mL)	Post‐autoclave calculated (g/L)
TEST	536*	26.8	26.6 ± 1.1	532	25.0 ± 2.0	501
SENSE	0.008	10.4	9.3 ± 0.7	0.007	10.7 ± 0.4	0.008

* Calculated applying theoretical adjustment of solute volume change.

## 4 CONCLUSIONS

The RP‐ UHPLC/MS and UHPSFC/MS assays developed here deliver fast, robust and quantitative analysis of homemade Bitrex™ and sodium saccharin solutions. Calibration curves acquired using SIM methods over the linear response range for each instrument give R^2^ values >0.99. If UHPSFC/MS is not available, an alternative RP‐UHPLC/MS method could be utilized providing that the calibration and sample solutions are made up using water rather than methanol as a solvent.
